# Promises, Pitfalls, and Basic Guidelines for Applying Machine Learning Classifiers to Psychiatric Imaging Data, with Autism as an Example

**DOI:** 10.3389/fpsyt.2016.00177

**Published:** 2016-12-01

**Authors:** Pegah Kassraian-Fard, Caroline Matthis, Joshua H. Balsters, Marloes H. Maathuis, Nicole Wenderoth

**Affiliations:** ^1^Neural Control of Movement Lab, Department of Health Sciences and Technology, Swiss Federal Institute of Technology, Zurich, Switzerland; ^2^Seminar for Statistics, Department of Mathematics, Swiss Federal Institute of Technology, Zurich, Switzerland

**Keywords:** ABIDE, classification, autism spectrum disorder, psychiatric disorders, machine learning, RS-fMRI

## Abstract

Most psychiatric disorders are associated with subtle alterations in brain function and are subject to large interindividual differences. Typically, the diagnosis of these disorders requires time-consuming behavioral assessments administered by a multidisciplinary team with extensive experience. While the application of Machine Learning classification methods (ML classifiers) to neuroimaging data has the potential to speed and simplify diagnosis of psychiatric disorders, the methods, assumptions, and analytical steps are currently opaque and not accessible to researchers and clinicians outside the field. In this paper, we describe potential classification pipelines for autism spectrum disorder, as an example of a psychiatric disorder. The analyses are based on resting-state fMRI data derived from a multisite data repository (ABIDE). We compare several popular ML classifiers such as support vector machines, neural networks, and regression approaches, among others. In a tutorial style, written to be equally accessible for researchers and clinicians, we explain the rationale of each classification approach, clarify the underlying assumptions, and discuss possible pitfalls and challenges. We also provide the data as well as the MATLAB code we used to achieve our results. We show that out-of-the-box ML classifiers can yield classification accuracies of about 60–70%. Finally, we discuss how classification accuracy can be further improved, and we mention methodological developments that are needed to pave the way for the use of ML classifiers in clinical practice.

## Introduction

Neuroimaging has substantially advanced our understanding of the perturbed neural mechanisms underpinning psychiatric disorders. However, the integration of neuroimaging tools into clinical practice has so far been limited, partly because it is unclear which information revealed by these tools is relevant for diagnosis and treatment decisions. To date, diagnosis focuses on behavioral manifestations, even though this approach is often time consuming, requires extensive experience and needs to be performed by a multidisciplinary team of specialists trained in the use of behavioral assessment instruments (1). Taking autism spectrum disorder (ASD) as an example of a psychiatric disorder, its current gold standard diagnosis is based on behavioral assessment instruments such as the Autism Diagnostic Interview-Revised [ADI-R; (2)] and the Autism Diagnostic Observation Schedule [ADOS; (3)].

Applying classification methods from modern statistics and Machine Learning to neuroimaging and/or behavioral data might increase diagnostic accuracy and speed up the diagnostic process. The datasets encountered in neuroimaging settings are often high-dimensional (large number of variables), and sample sizes are relatively small even if data repositories are used (4). Therefore, many ML approaches incorporate feature selection strategies (either based on expert knowledge or applying automatic feature-engineering methods), which allow them to reduce dimensionality (see Feature Calculation and Feature Selection for a more detailed discussion of variable selection techniques). Moreover, ML classifiers can detect biomarkers for the disorder, subtypes of the disorder, and comorbidities. Hence, ML classifiers have the potential to aid the integration of neuroimaging data into clinical practice.

ML classifiers are algorithms that predict for each subject to which class [here ASD versus typically developed (TD)] it belongs, based on data (here neuroimaging information). ML classifiers first learn how to separate the classes based on data where the class labels (here ASD and TD) are provided to the classifiers. This is called the training stage. Subsequently, the trained classifiers can apply the learned separation rule to unseen data to predict the corresponding labels. In our setting, this means that the classifier is applied to neuroimaging data of new subjects to predict whether or not they have ASD.

We will present the entire classification pipeline using multisite resting-state fMRI (RS-fMRI) data from the Autism Brain Imaging Data Exchange (ABIDE) repository (5). Even though much current knowledge about the pathophysiology of psychiatric disorders was derived *via* task-based neuroimaging paradigms, spontaneous or resting-state fluctuations in the blood oxygenation level-dependent (BOLD) signal are increasingly employed to investigate neural connectivity and identify biomarkers of psychiatric disorders. The underlying hypothesis is that ASD and TD subjects can be distinguished by differences in functional connectivity, measured as correlations between these spontaneous signal fluctuations (5). RS-fMRI is promising as a clinical tool, because it is relatively fast to acquire (6, 7), task-free (thus requiring minimal cooperation of the patient), and data can be easily combined across multiple scanning centers to generate large databases such as ABIDE. Basing classification on such a large, multicenter database helps to capture the heterogeneity of the psychiatric disease and generalize results across multiple fMRI setups. Furthermore, it is well established that networks of correlated brain activity can be identified during rest, and that the principle anatomy of these networks is preserved across individuals, which is advantageous for dimensionality reduction, as desirable for classification (8). Finally, RS-fMRI can be used for the prediction of a variety of diseases such as depression, schizophrenia, or Parkinson’s disease (9, 10).

Site-specific effects, however, might introduce variability into the data that makes prediction of the disorder more difficult. Previous RS-fMRI ASD classification studies have seen a considerable drop in classification accuracy when switching from single-site to multisite data (11, 12). Nevertheless, recent multisite ASD classification studies based on RS-fMRI have shown that the disorder can be predicted with accuracies between 60 and 79% (12–14).

Using ASD as an example, the objective of the present article is to provide a lucid and praxis-oriented tutorial that enables a wider audience to use publicly available ML classifiers for the prediction of psychiatric diseases. We begin by introducing basic concepts and discussing important methodological choices that can impact classification accuracy or sensitivity. We then present and compare the classification results for several classifiers, based on RS-fMRI data of 154 subjects from the ABIDE database. The presented classifiers are commonly used for the classification of neuroimaging data (4). Furthermore, their implementation is provided by MATLAB, such that they can be easily employed by the reader. Finally, we highlight potential pitfalls and challenges that can occur at different stages of the classification process. We also provide the data and the MATLAB code for all discussed pipelines. Although this tutorial focuses on ASD classification, it can also be used for the classification of other psychiatric disorders based on RS-fMRI data.

## Data

We illustrate all methods in this paper on connectivity matrices computed from the ABIDE dataset (5). In this section, we explain how the connectivity matrices are obtained from the raw data.

### Feature Calculation

**Figure 1:**
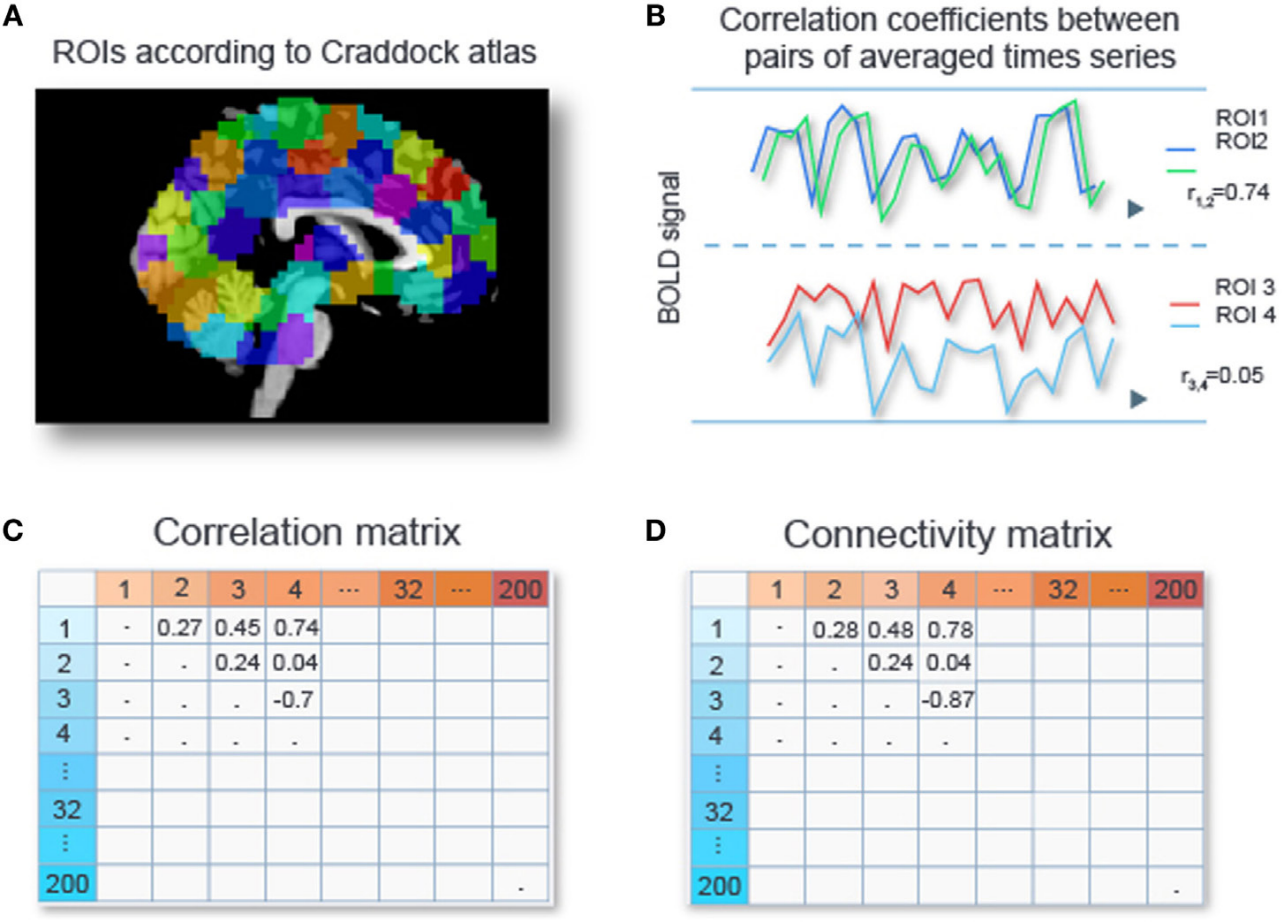
**Calculating connectivity features**. Two hundred ROIs are determined from the Craddock atlas (15) **(A)**. For each subject, the time series of voxel intensities are averaged over voxels with an ROI, and Pearson correlation coefficients are computed for all pairs of averaged time series **(B)**, resulting in a 200 × 200 correlation matrix **(C)**. Each entry in the correlation matrix is transformed with Fisher’s *z*-transform to obtain connectivity values **(D)**.

Data sets from fMRI studies often possess a large number of predictors (or features) relative to the number of data samples (4). For our particular data set we are left with time series for circa 50,000 voxels per subject after removal of signals of no interest such as head movement, respiration, and scanner-related artifacts (see Preprocessing of the fMRI Data in Appendix A1). A subject’s connectivity pattern can be estimated by calculating correlations between the time series for all its pairs of voxels. These correlations can be used as predictors (“features” in Machine Learning jargon) for the classifiers. Due to the large number of voxels per subject, fMRI studies often possess such a large number of predictors (or features) relative to the number of data samples (4). This can cause classifiers to adapt to peculiarities of a specific dataset (“overfitting”) and results in poor generalizability. Hence, reducing data dimensionality for alleviating the problem of overfitting is crucial, especially in settings where restricted amount of data are available. Various dimensionality reduction techniques are applicable to RS-fMRI data sets, and they will be discussed in detail in Section “Feature Selection.” Out of these various options, we chose to perform an initial dimension reduction by averaging time series of voxels within regions of interests (ROIs), since time series of voxels within a ROI tend to be highly correlated. Craddock et al. (15) showed that partitioning the cerebral cortex into 200 or 1000 spheres provided more homogeneous time series within ROIs than using parcellations offered by gross anatomical atlases. Based on this, we work with 200 ROIs from the Craddock atlas. We compute the average time series for each ROI, and then compute the correlations between all pairs of averaged time series, yielding a 200 × 200 correlation matrix. Next, we apply Fisher’s *z*-transformation (16) to each entry in this matrix, yielding a 200 × 200 connectivity matrix. Since this matrix is symmetric, this leaves us with 19,900 unique features per subject. Figure 1 gives a schematic overview of the described procedure.

### Subject Selection

We exclude underrepresented subjects including: female subjects (12%), subjects older than 40 years (8%), and those with an intelligence quotient (IQ) below 80 (8%). This also reduces the complexity in our data set; however, it might be worthwhile to investigate the entire spectrum in future classification approaches. We also exclude subjects with strong artifacts due to head movements (see Preprocessing of the fMRI Data in Appendix A1).

We balance the data per site, meaning that we take the same number of ASD subjects as TD subjects per site. Furthermore, we ensure that the 2 resulting classes (TD and ASD) with 77 subjects each are similar on average with respect to IQ, age, and head movements. This prevents the classifier from separating classes based on these variables instead of the class labels. If one of the classes for instance contains many more low-IQ subjects than the other class, the classifier could deliver optimal results by learning to separate between low and higher IQ values. The application of this classifier in a clinical setting could potentially produce false positives (FPs) by labeling low-IQ individuals without ASD as having ASD, or false negatives (FNs) by labeling high IQ individuals with ASD as TD. Therefore, while it is important to build classifiers using heterogeneous datasets that reflect real-world populations, it is also important at this early stage to match datasets in order to confirm that classifiers are not distinguishing class labels using variables other than RS-fMRI connectivity. In the future, it might be important to classify ASD not only in comparison to TD but also in comparison to other neurodevelopmental pathologies. Female subjects were excluded from our data set because the underlying neuropathology might differ dramatically between the sexes causing highly deviating rs-FMRI connectivity (17). Thus, females might potentially represent an independent subclass of ASD, but we had too few data points in our sample to investigate this issue and, therefore, focused on males only. For an in-depth analysis of the connection between classification accuracy, sample size, and data heterogeneity in classification studies of neuroimaging data, we refer the reader to Schnack and Kahn (18). Schnack and Kahn conclude that in general classification studies with smaller data sets might display higher classification accuracies due to the higher heterogeneity of the larger data set and point to the importance of taking sample sizes into account when comparing the classification results of different studies.

The balancing is achieved by under-sampling (i.e., including fewer subjects than available in the original dataset), leaving us with a total of 154 data samples, 77 for ASD and 77 for TD. Working with balanced classes has the advantage that the classifier’s performance can be assessed easily by classification accuracy – the number of correctly classified data samples over all data samples.

## Feature Selection

Feature selection refers to the selection of a subset of the available features (here connectivity values, i.e., entries of the connectivity matrix) for classification. Proper feature selection can enhance classification accuracy, facilitate visualization of the data, and lead to faster classification (19, 20). An alternative to feature selection is feature extraction. Feature extraction methods transform the original features into a lower dimensional feature space (19), using for example principal component analysis. Thus, rather than selecting certain features, one works with selected combinations of the original features. Changing the feature space however can complicate the interpretation of results. Hence, feature selection might be preferable if interpretability is pivotal.

In fMRI-based analyses, feature selection or extraction is especially critical, since the data are usually very high dimensional, even after voxels are summarized to ROIs. After having performed feature extraction by summarizing voxels to ROIs (see Feature Calculation), we perform feature selection to further reduce the dimensionality of the data and increase classification accuracy. Methods for feature selection are typically divided into filter methods and wrapper methods (21).

### Filter Methods

Filter methods select features based on a given statistical criterion, and only features with high scores for the criterion are retained. An example of such a criterion is the *t*-statistic from a two-sample *t*-test, where high scores indicate that the given feature effectively separates the classes (11).

Filter methods are usually computationally inexpensive (19, 22). However, most filter methods are univariate, meaning that they assess each feature individually. Hence, they do not account for feature dependencies (22). Moreover, they do not take interactions with the classifier into account. In other words, classifier performance is not part of the selection criterion (22).

### Wrapper Methods

Wrapper methods employ the classifier to determine an optimal feature subset (23). In particular, the classifier is applied and evaluated using different feature subsets, and the best performing feature subset is selected. An example of such a procedure is recursive feature elimination (RFE). RFE first applies the classifier using the full feature set, and in each following step the least useful features are discarded.

Wrapper methods have a higher computational cost due to their iterative approach, but they account for dependencies between different features and are naturally tailored to the classifier they are combined with. Plitt et al. (13) report a successful application of RFE combined with linear support vector machines (SVMs) to determine which features are most predictive for ASD classification.

A recommendable practice consists of an initial feature reduction with a filter method, followed by a wrapper method on the reduced feature set (22). More details on feature selection can be found in Guyon and Elisseeff (19).

## Performance Assessment

Classifiers can be assessed by different assessment measures, such as accuracy, sensitivity, and specificity. Crucially, they should be assessed on different data than the data on which they were trained. We start by explaining the important distinction between test accuracies and training accuracies [see also James et al. (24), Chapter 2.2].

### Test Accuracy versus Training Accuracy

We randomly divide the data into two parts, a training set and a test set, each consisting of a subset of the samples. The classifier is trained on the training set, meaning that the classifier learns to separate the classes optimally, based on the features and labels of the training set.

Applying this learned classification rule to the features of the training set, pretending we forgot the labels, results in predicted labels for all samples in the training set. These predictions can be compared to the true labels of these samples. The training accuracy measures the percentage of correct predictions on the training set, i.e., the number of correctly predicted labels over the number of samples in the training set.

It is important to note that this training accuracy is overly optimistic, since it evaluates the classifier on the same data on which it was trained. In practice, however, we are interested in the performance of the classifier on new and unseen data. For instance, we would be interested in a classifier’s performance for incoming patients and not for already diagnosed patients. To mimic this situation, we can apply the classifier that was trained on the training data to the features of the test set. Comparing the resulting predicted labels to the true labels of the test set leads to the notion of test accuracy (as opposed to training accuracy mentioned above), which is the percentage of correct predictions on the test set. Since the test set is from the same distribution as the training set, but independent from it, this allows a fair estimation of the classifiers’ generalization performance on unseen data from the same distribution.

### Accuracy, Specificity, and Sensitivity

The accuracy summarizes the overall performance of the classifier by measuring the percentage of correct predictions among all samples that have been classified. To describe more detailed performance measures, the following terminology is needed.

Samples that are correctly classified as having a condition (here ASD) are called true positives (TPs). Samples that are correctly identified as not having the condition (here TD) are called true negatives (TNs). Classification errors can occur in two ways. If a sample without the condition is classified as having the condition, it is called an FP. If a sample with the condition is classified as not having the condition, it is called a FN.

Using the notation #TP, #TN, #FP, and #FN for the number of TPs, TNs, FPs, and FNs, it follows that $\text{accuracy}=\frac{\#\text{TP}+\#\text{TN}}{\#\text{TP}+\#\text{FP}+\#\text{TN}+\#\text{FN}}.$ The so-called sensitivity or TP rate is the ratio of correctly classified subjects with the condition over all subjects with the condition, i.e., $\text{sensitivity}=\frac{\#\text{TP}}{\#\text{TP}+\#\text{FN}}\text{.}$ The so-called specificity or TN rate is the ratio of correctly classified subjects without the condition over all subjects without the condition, i.e., $\text{Specificity}=\frac{\#\text{TN}}{\#\text{TN}+\#\text{FP}}.$ A common way to summarize the absolute number of TPs, TNs, FPs, and FNs is a contingency table (25).

For unbalanced datasets, accuracy may be misleading. For instance, suppose that a classification of two-class data is performed on a 9:1 class-size ratio (i.e., 9 ASD to 1 TD). Then, the performance of the classifier on the larger set will count nine times as much as the performance on the smaller set. Hence, high classification accuracy can simply mean that the classifier is by default predicting the larger class (26). For binary classifiers, this problem can be alleviated by combining the classification with sampling techniques for creating classes of equal size. Under-sampling techniques for instance create classes of equal size by sampling from the larger class as many data samples as the smaller class possesses, while over-sampling methods sample from the smaller class until the size of the larger class is attained (27). Another option is to use performance measures based on sensitivity and specificity, such as for instance a comparison of the TP and FP rates as functions of classifier parameters in a receiver operating characteristic (ROC) curve (26, 28, 29). The curve graphically illustrates the TP rates (*y*-axis) and the FP rates (*x*-axis) as a function of the classifiers parameters – an ideal classification would yield a TP rate of 1 and a FP rate of 0, whereas random guessing should yield points representing an equal rate of TP and FP, respectively. Brodersen et al. (30) also introduce the concept of a “balanced classification accuracy”: this balanced accuracy is the average of the TPs over the positives (here: ASD) and the TNs over the negatives (here: TD). Alternatively, accuracy measurements for unbalanced datasets can also be embedded in a probabilistic framework, where a confidence interval for the accuracy is calculated.

### Cross-Validation

**Figure 2:**
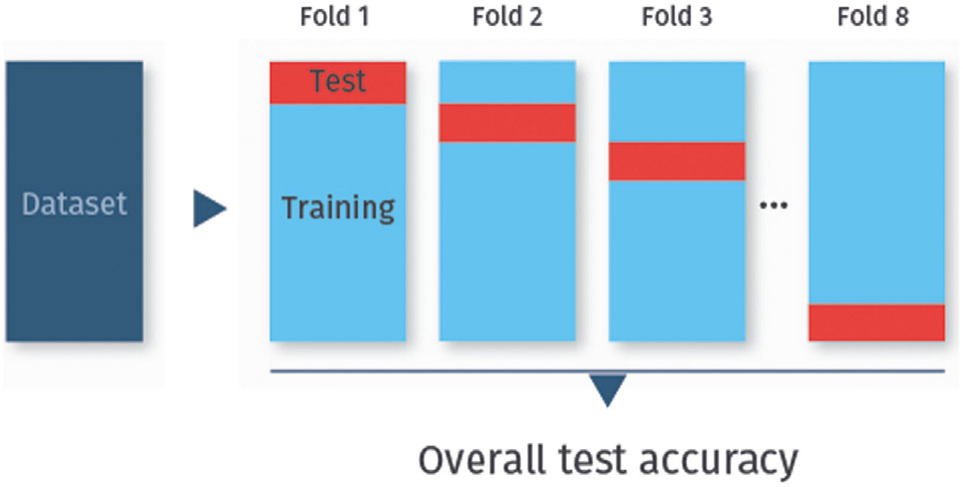
***k*-fold cross-validation**. In *k*-fold cross-validation, the data are randomly split into *k* folds (here eightfolds). Each fold is used once as test set, while the remaining data are used for training.

To determine the test accuracy, sensitivity, or specificity, the data are usually split not only once into a training set and a test set, but repeatedly. In particular, the data are randomly split into *k* disjoint sets of approximately equal size, called folds. Each fold is used once as a test set, while all other folds combined then serve as the training set. This procedure is called *k*-fold cross-validation [(24), Chapter 5.1]. Cross-validation is the method of choice for assessing the classifier’s performance on previously unobserved data. Figure 2 visualizes eightfold cross-validation as an example.

The most common cross-validation schemes are leave-one-out cross-validation (LOO cross-validation), where *k* equals the number of data samples, and 10-fold cross-validation (*k* = 10). For most data sets, 10-fold cross-validation is a good compromise with regard to bias (the expected difference of the classifier’s prediction as compared to the true class-membership) and variance (the variability of the classifier’s prediction for one data samples), and is hence widely used (31).

**Figure 3:**
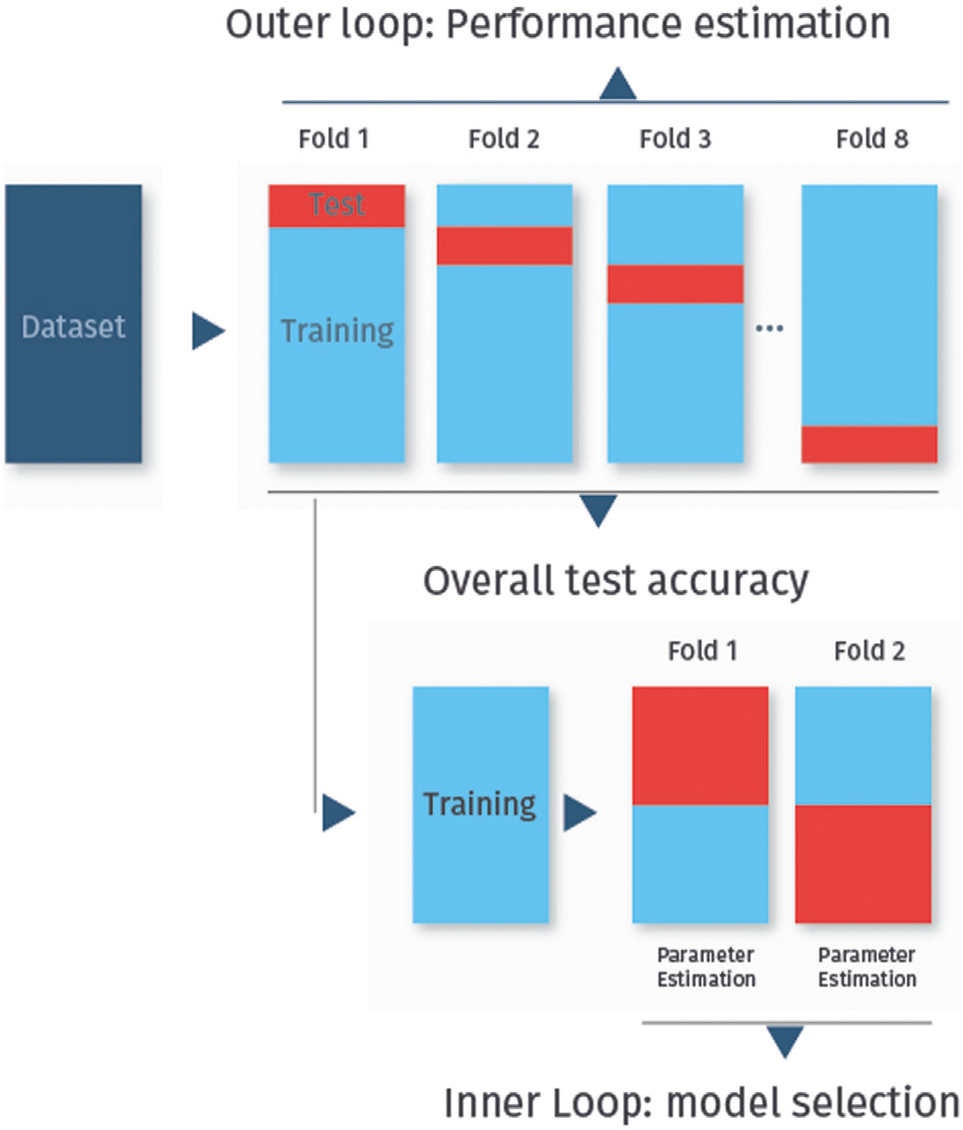
**Nested cross-validation**. In nested cross-validation, the data are split twice into several folds. In this figure, we have eightfold outer cross-validation to determine the classifier performance. In the inner twofold cross-validation, the training set is divided again for feature selection or to determine optimal tuning parameters for the classifier.

Cross-validation can also be used in combination with feature selection or the selection of tuning parameters like the penalization parameter in lasso-regularized logistic regression (see Lasso-Regularized Logistic Regression). In this case, nested cross-validation must be used to avoid optimistically biased performance estimates (32). The idea is to start with a regular *k*-fold cross-validation, called the outer loop, to assess the final classifier performance. As before, each fold is used once as the test set, while all other folds are then used as the training set. Each training set is then randomly split again into several folds, in the so-called inner cross-validation loop, which is used for feature selection or tuning parameter selection. Thus, the training and test sets from the inner loop are used to try out different feature subsets or classifier tuning parameters, and the best performing classifier from this level is then applied to the test set from the outer loop. The process of nested cross-validation is shown in Figure 3.

Ideally, after cross-validation the optimized classifier is applied to an entirely new and independent data set (the so-called validation set). Classification performance on the fresh data from the validation set is a better measure for how well the classifiers generalize [(24), Chapter 5], and hence of how well it diagnoses yet unseen subjects. However, performing this step assumes the availability of enough data.

## Classifiers

ML classifiers allow the multivariate analysis of many features together, thereby allowing for good predictive performance (33–35). This stands in contrast with most of the traditional fMRI analysis approaches (so-called mass univariate methods), which rely on single features (35).

**Figure 4:**
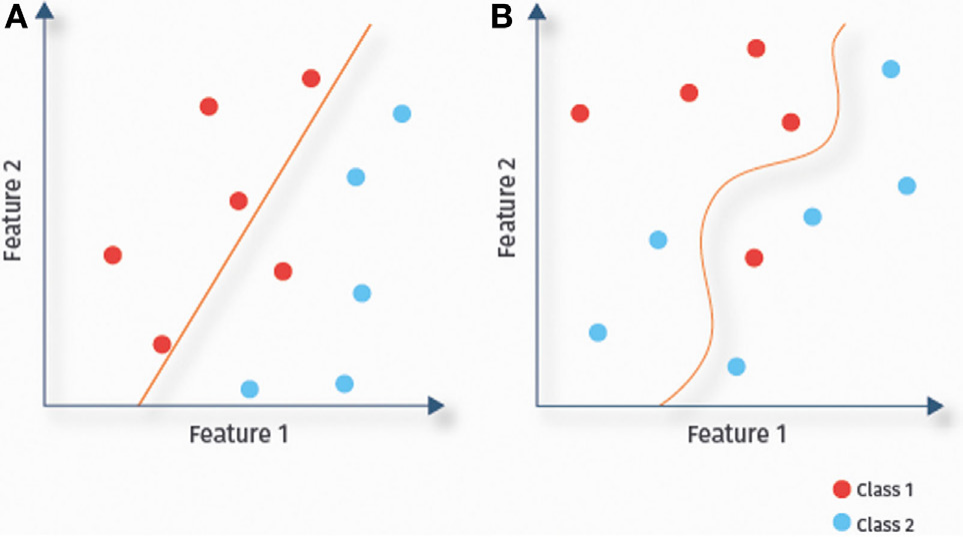
**Linear versus non-linear classification**. Illustration of the separation of data samples of a two-class problem by fitted decision boundaries. Depending on the shape of the decision boundary, classifiers are categorized as linear **(A)** or non-linear **(B)**.

A classifier uses the available data to determine a decision boundary to separate classes (here ASD and TD) within the multidimensional feature space. Classifiers are called linear or non-linear, depending on the decision boundary being linear or not. In linear classification (Figure 4A), each feature is associated with a particular weight, which reflects its relevance for the prediction (21), allowing for a straightforward interpretation of results. Due to their lower complexity, linear classifiers are also less prone to overfitting than some non-linear classifiers. This might explain the success of linear classifiers for RS-fMRI-based ASD prediction (13, 14, 36). In the remainder of this section, we discuss several linear classifiers [(regularized) logistic regression, linear SVMs, and linear discriminant analysis (LDA)], as well as several non-linear classifiers (Figure 4B), including Gaussian naïve Bayes (GNB), kernel SVMs, and probabilistic neural networks.

The choice of a well-suited classifier depends on various factors, including the dimensions of the dataset, the feature selection method, the required classification speed, and the statistical properties of the data. For example, if the dataset contains strongly correlated features, the performance of some classifiers such as GNB can degrade. However, a suitable feature selection method can alleviate this problem (21). Other classifiers, such as for instance SVMs, tend to perform well even without any previous feature selection and when the features are strongly correlated [(20, 24), Section 9.2.2].

In high-dimensional settings, better classification performance and a lower risk of overfitting can also be achieved by imposing constraints on the statistical model. This is called regularization [(24), Chapter 6.2]. In the case of regression, we might for instance want to restrict the magnitude of the regression coefficients to avoid overfitting to noisy data (37). This can serve as an inbuilt feature selection procedure where uninformative features are removed from the classification process by setting the associated regression coefficients to zero (as in Section “Lasso-Regularized Logistic Regression”).

In the remainder of this section, we present several well-known classifiers and discuss their assumptions and properties. All presented classifiers are pre-implemented, easy-to-use, and commonly used for the classification of RS-fMRI data (13, 14, 36). We refer to James et al. (24), Hastie et al. (38), and Bishop (39) for more details on the presented classifiers. For an excellent brief introduction to the formal background of these algorithms and a subsequent discussion of their application to brain imaging data, we refer the reader to Lemm et al. (40).

### Logistic Regression

Logistic regression is a type of regression where the predicted class variable is binary. This fits our setting, since our classes can be labeled as 1 and 0 (ASD and TD). Logistic regression can be viewed as a special case of a generalized linear model, where the log odds is modeled as a linear function of the predictors. A convenient property of this model is that the sizes and signs of the estimated coefficients have a clear interpretation. Please see Chapter 4.3 of James et al. (24) for details.

### Lasso-Regularized Logistic Regression

Important regularized variants of logistic regression are ridge logistic regression and lasso-regularized logistic regression. Due to our high-dimensional data set, we focus here on lasso since as mentioned this method removes uninformative features by setting the associated regression coefficients to zero. Computationally, regularization is performed by introducing a regularization parameter, which can be optimally chosen *via* cross-validation.

### Support Vector Machines

The basic idea of linear SVMs is to construct an optimal linear decision boundary that is maximally far from the data samples of the two classes. SVMs belong to the category of regularized predictors – a regularization term determines to what extent misclassification of data samples is accepted. Not allowing for any misclassification might lead to poor generalization of the classifier, due to overfitting to a particular data set [(24), Chapter 9.2.2].

**Figure 5:**
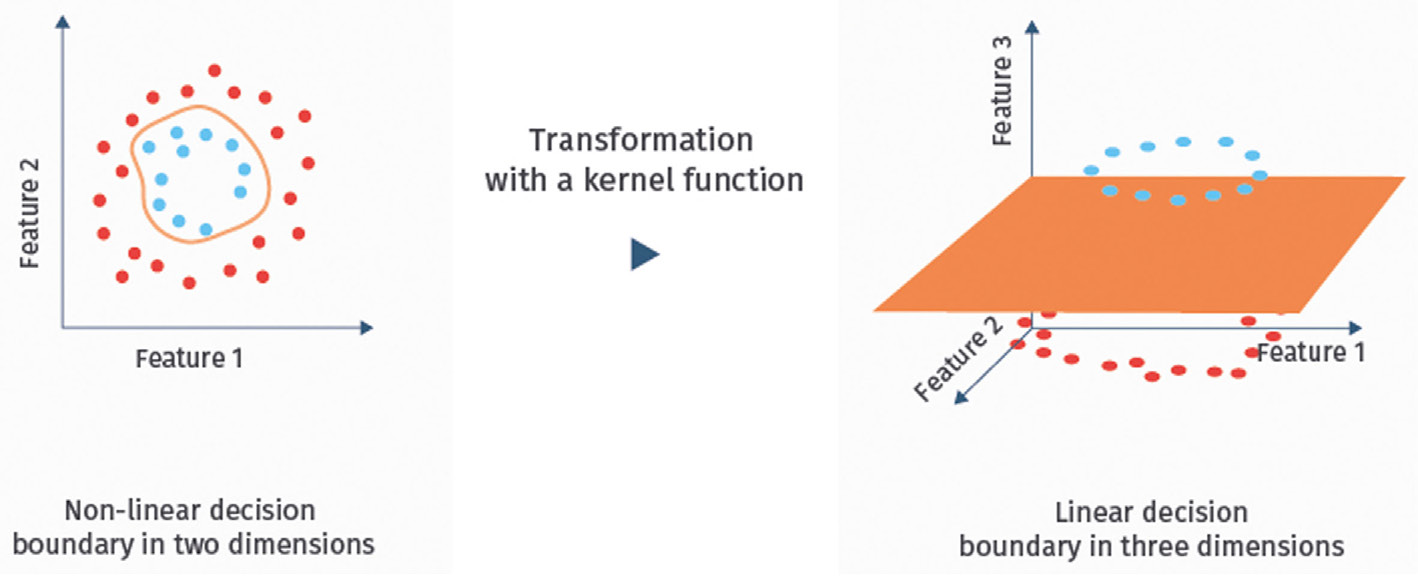
**Example of a kernel mapping**. The left hand side shows a dataset that cannot be separated linearly in the two feature dimensional space. The right hand side shows a three-dimensional embedding, where linear separation is possible.

It is well-known that SVMs can handle noisy, correlated features and high-dimensional data sets well [(24), Chapter 9.2.2; (41)]. Hence, they have become one of the most successful classifiers of the recent years, also for the classification of fMRI data (13, 21). If the data are not linearly separable in the original feature space, one can map them with a so-called kernel function into a higher dimensional space to achieve separability [(24), Chapter 9.3.2]. The resulting classifier is then called kernel SVM. An illustration of this approach is given in Figure 5. Linear SVMs, however, have so far been more successful for the classification of ASD based on RS-fMRI data than kernel SVMs (13).

### Probabilistic Neural Networks

**Figure 6:**
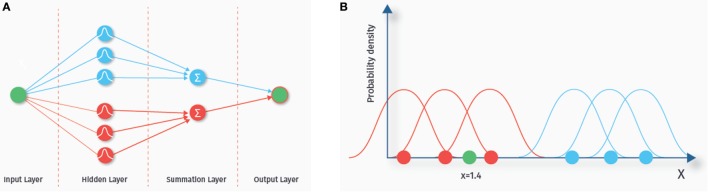
**Probabilistic neural network**. The left hand side **(A)** shows the network architecture. Each feature is represented by an input node. Each sample in the training set is represented by a node in the hidden layer. The nodes of the hidden layer evaluate the density value of a new, yet to classify data sample. The nodes in the summation layer sum up the density values for each class. Finally, the output layer outputs the class with the highest estimated membership probability. On the right side **(B)**, a one-dimensional example data set is shown. The blue and the red dots represent training data from two different classes. A chosen probability distribution, in our example a Gaussian distribution, is centered at each of the data points of the training set. The green dot (*x* = 1.4) is a new data point we want to classify. In our example, the density values of the Gaussians from the blue class are small at the location of the green dot, whereas the density values of the red class are higher, indicating that the green dot is more likely to belong to this class.

The term neural network comes from the fact that the structure of these classifiers (depicted in Figure 6A) is thought to somewhat resemble biological neuronal networks. A probabilistic neural network consists of various layers, each containing a number of nodes (42, 43). We explain the basics of the classification algorithm with the one-dimensional example data depicted in Figure 6B. Nodes in the first layer of the probabilistic neural network are called input nodes. This layer contains as many nodes as there are features. A one-dimensional example data set is shown in the right panel (corresponding to one input node). The blue and the red dots represent training data from two different classes. The number of these data samples determines how many nodes the next “hidden” layer contains. A chosen probability distribution, in our example a Gaussian distribution, is centered at each of the data points of the training set. The green dot (*x* = 1.4) is a new data point we want to classify. Each of the hidden nodes evaluates the density value of its Gaussian for the green dot. In our example, the density values of the Gaussians from the blue class are small at the location of the green dot, whereas the density values of the red class are higher, indicating that the green dot is more likely to belong to this class. Next, the summation layer outputs the sum of these density values for each class. The last layer then consists of a single node that outputs the label of the class with the highest membership probability. Probabilistic neural networks are known to be relatively fast and have been used previously to predict ASD based on ABIDE RS-fMRI data (44).

### Linear Discriminant Analysis

Linear discriminant analysis assumes that the features (in our case entries of the connectivity matrix) within each class (in our case TD or ASD) follow a multivariate normal distribution, with a common covariance matrix and different mean vectors. The class means and the common covariance matrix can then be estimated from the data, leading to two estimated multivariate normal densities. Then, for a new data sample *x*, the estimated densities are evaluated, and the sample is assigned to the class with the highest estimated density.

Alternatively, LDA can be viewed as seeking a one-dimensional projection vector that maximizes the ratio of between class variance over within class variance. In this sense, the multivariate normal assumption is not necessary. For further reading, we recommend Duda et al. (45).

### Gaussian Naïve Bayes

The GNB classifier assumes that the features of each class follow a multivariate normal distribution with an arbitrary mean vector and a diagonal covariance matrix (with arbitrary entries on its diagonal). The diagonal covariance matrix entails the assumption that the features within each class are independent, and that they can have arbitrary variances. During training, the means and variances are estimated. Subsequently, like for LDA, a new data point is assigned to the class that is most likely to have generated it.

It has been shown that GNB classifiers can operate reasonably well even if the independent features’ assumption is not fulfilled, but its performance degenerates when the correlations are very strong (46). GNB classifiers are easy to implement, as well as fast.

## Applying the Classifiers to the Abide Dataset

We now apply feature selection and several classifiers to our multisite ABIDE dataset. The inputs to the classification pipeline are the 77 ASD and 77 TD connectivity matrices (hence 154 data samples in total), where each connectivity matrix consists of 19,900 features.

With the exception of lasso-regularized logistic regression, we perform feature selection to reduce the number of features and hence the risk of overfitting. Out of many different possibilities for feature selection, we use a simple and fast filter method called thresholding. For each feature (i.e., each connectivity value), we calculate the absolute difference between the class means (ASD versus TD means). The feature is selected if this absolute difference is larger than a threshold value *t*. The assessed range of *t* values is between 0 (resulting in all 19,900 features selected) and 0.18 (resulting in about 7 features selected). Mostly, a *t* value of around 0.15 was selected, corresponding to around 40 features.

The performance of each classifier is assessed by nested cross-validation: 10-folds are used in the outer cross-validation loop for the performance estimation, and 10-folds are used in the inner cross-validation loop to determine the optimal threshold value *t* for feature selection, or the optimal regularization parameter for lasso-regularized logistic regression. For all other classifiers, MATLAB’s default setting is used for the tuning parameters.

We also assess statistical significance of each classification procedure with respect to the null hypothesis of random guessing, by means of permutation testing (47). The importance of significance testing is further elaborated in Section “Comparison of Classification Accuracy to Chance Level.” Permutation testing is detailed in Section “Tests for Classification Significance” in Appendix A2.

Table 1 summarizes the performance of the classifiers in terms of test accuracy, sensitivity, and specificity. We will see in Section “Building Classifiers for Multisite Data” that removing site effects will lead to even higher classification accuracies. All classifiers were better than random guessing *p* < 0.001.

**Table 1:** **Overview of classifier performances**.

Classifier	Accuracy	Specificity	Sensitivity	*p*-Value
LR	0.58	0.59	0.57	0.009
LassoLR	0.58	0.57	0.56	0.009
SVM	0.63	0.64	0.62	0.007
PNN	0.58	0.57	0.59	0.009
LDA	0.57	0.58	0.56	0.009
GNB	0.61	0.63	0.62	0.008

*LR, logistic regression; LassoLR, lasso-regularized logistic regression; SVM, linear support vector machine; PNN, probabilistic neural network; LDA, linear discriminant analysis; GNB, Gaussian naïve Bayes*.

*This table provides an overview of the performances of the classifiers discussed in Section “Classifiers.” Listed are the test accuracy, specificity, and sensitivity. Nested cross-validation was performed for all classifiers, with 10-folds for both the outer and the inner loops*.

## Pitfalls and Challenges

### Cross-Validation and Feature Selection: The Peeking Problem

Even when applying out-of-the-box classifiers for the classification of psychiatric disorders, important challenges and pitfalls in the analysis pipeline remain. One pitfall is given when feature selection or selection of tuning parameters is performed on the full data, i.e., on data samples from both the training and the test set.

To illustrate this, we simulated high-dimensional data as follows. We generated 80 data samples for each of 2 classes, by randomly sampling 20,000 features from independent standard normal distributions for each data sample. Since the labels are not associated to the features, the true test accuracy of a method is at best 50%. If, however, we use the entire dataset to select the features with a mean difference above a threshold value *t* = 0.10, classification with a linear SVM yields a test accuracy of 91%. If the feature selection is correctly applied using only the training set, the classification accuracy is between 45 and 56%. This pitfall is referred to as peeking or double-dipping. Our simulation illustrates that peeking is a serious problem that can result in severely biased accuracies. Assuming a binomial distribution (see Tests for Classification Significance in Appendix A2), a classification accuracy of above 62.5% is deemed significant for a *p*-value below 0.01.

Systematic reviews of research articles show that double-dipping is still common. Kriegeskorte et al. (48) report that at least 42% of fMRI studies published in high impact journals during 2008 were affected (48, 49). The bias caused by double-dipping is especially large for data sets with few data samples and a large number of features (50), as is often the case for neuroimaging data sets.

It has to be noted that if cross-validation is performed to find optimal tuning parameters for the classifier, the performance of the optimized classifier has to be evaluated on a new data set (nested cross-validation). Otherwise, the performance evaluation can again be optimistically biased (32). Since different performance estimation procedures can have a large impact on the classification results, including a detailed description of these should be standard procedure in publications.

### Building Classifiers for Multisite Data

The use of multisite data poses a challenge for the classification of ASD, since site-specific variability makes it more difficult for classifiers to detect information that is important for the prediction of the disorder. Previous ASD classifiers that were tailored to RS-fMRI data from a single site (11) degraded markedly in performance when applied to multisite data (12). The classification accuracy dropped from roughly 80 to 60% (12).

To reduce site-induced variability in the data set, a first step is to take linear site effects into account. Accounting for linear site effects can be done by using a *z*-transform within each site. Thus, for a subject *k* from a given site, we compute the standardized *i*-th feature as follows: $\frac{\left(i\text{-th feature of subject }k\right)-\text{mean}\left(i\text{-th feature of site}\right)}{\text{standard deviation}\left(i\text{-th feature of site}\right)}$.

We took the so far best performing classifier – the support vector machine – and applied it to the data set after this standardization was performed. The resulting classification accuracy increased from 63 to 68%. Note that the double-dipping problem has to be considered for standardization as well: standardization must be done for training and test set independently, and in both cases, the mean and the SD from the training set have to be used in order to avoid double-dipping.

Another possibility to assess the generalizability of the classifier to data from different sites is to form training and test sets from different sites. An example of this approach is leave-one-site-out cross-validation, where the test set contains data from a site that has not been used in the training set (36). The suggested *z*-transformation to remove linear effects is only a first step in the removal of site effects. In a next step, it will be worthwhile to balance the data from each site for variables such as sex, age, or IQ. Furthermore, we would like to point the reader to elaborated methods to remove even complex site effects, developed in microarray studies where data stems commonly from many different sites. Gagnon-Bartsch et al. (51) and Leek and Storey (52) both provide methods in which unwanted factors (as, for instance, site) are estimated from the data, and subsequently included into the design matrix of a regression model, or unwanted variation is modeled as part of an error term.

### Small Sample Size

Several challenges can emerge when the number of features strongly exceeds the number of data samples, as is the case in the given setting. A first problem is the high risk of overfitting. A small but possibly complex data set can evoke an idiosyncratic fit with poor generalizability.

A second pitfall concerns the detection of the most predictive features. Detecting such features can be desirable to determine the functional networks associated with them. Highly predictive features can also be correlated with behavioral assessments of autism [as, for instance, the Social Responsiveness Scale (13)]. This correlation can be employed to assess if the classification delivers medically interpretable results and accounts for continuous symptom manifestation beyond binary separation (13). The difficulty, however, is that different cross-validation rounds and different classification methods will rank other sets of features as most predictive. This variability is especially high in the case of small-sized data sets and requires careful neuroscientific interpretation. In these instances, it is recommended to use stable feature selection methods for small data sets to guarantee such robustness (22).

### Comparison of Classification Accuracy to Chance Level

It is common for neuroscientific studies to compare classification accuracies to chance level. Chance level is thereby the accuracy achieved assuming that it is equally likely for a data sample to fall in any of the existing classes. In the case of a balanced two-class problem, chance level classification accuracy would equal 50%, and for a balanced five-class problem it would amount to a classification accuracy of 20%. However, chance level accuracies are theoretical values derived for random guessing on data sets of infinite size. Although random guessing will approximate chance level accuracies if the data set is large enough, for small data sets as often encountered in neuroscientific studies, random classification can deliver accuracies strongly deviating from chance level. Combrisson and Jerbi (53) show that when applied to small data sets, various classifiers can achieve accuracies as high as 70% for a two-class problem where labels are uninformative and chance level is 50%.

Instead of comparing classification results to a theoretical chance level, parametric or non-parametric statistical tests can be applied where data size is taken into account (53). Parametric tests assume an underlying distribution for the data set, whereas non-parametric statistical tests work with minimal statistical assumptions (47). Appendix A2 explains these tests in more detail.

## Summary and Concluding Remarks

In this tutorial, we presented several standard Machine Learning classifiers and their advantages and disadvantages for the classification of ASD, based on multisite neuroimaging data. The presented classification pipeline for ASD served as an example for the classification pipeline of psychiatric disorders in general. The presented classifiers reached peak accuracies of around 60–70%. Given that the information used for classification was retrieved from neuroimaging data and not from the established behavioral markers, and that straightforward methods were used for the prediction, this prediction approach is worthwhile pursuing with more elaborated methods.

One reason for the nevertheless relatively low classification accuracies could be the variability in RS-fMRI data introduced from data collection at different sites. We saw that accounting for linear site effects can improve the accuracy. Accounting for non-linear site effects might increase accuracies further.

Several other steps in the classification pipeline could be enhanced as well. First, variations of these classifiers tailored for small but high-dimensional data sets might deliver better classification accuracies [see, for instance, the LDA classifier developed by Qiao et al. (54)]. Second, more sophisticated feature selection methods than simple thresholding might further boost classification accuracy. One may also consider alternative atlases to determine the ROIs (13). Furthermore, the use of structural brain data for the ML-based prediction of psychiatric diseases such as schizophrenia, ASD, or the classification between unipolar and bipolar depression based on such features has been shown to be fruitful (10, 55–58). Wolfers et al. (59) provide a literature survey on ML-based methods in psychiatric research and investigate the current state of translating these methods into clinical practice. In their review, they also investigate the imaging modalities used for classification and conclude that useful features can be extracted across different imaging modalities, and that different imaging modalities can achieve similar classification accuracies. Hence, in a next step, combining RS-fMRI data with data gained from other techniques might be beneficial.

It is also important to note that the labels (patient or control) used in the classification pipeline are attained through behavioral assessments. This means that the labels are noisy, i.e., we cannot be certain that the label is correct in every case, and hence classification accuracy is limited by the accuracy of behavioral assessments. Employing classification approaches that account for the noisy labeling might deliver superior results (60). An alternative is to apply unsupervised methods that do not require any class labels [(24), Chapter 10]. Clustering methods, for instance, can pool data samples into groups according to some notion of similarity. Deep learning offers the possibility of unsupervised feature selection: simple features are extracted by the algorithm in the lower layers and combined to more complex features in the later layers. Plis et al. (61) have shown that neuroscientifically meaningful features can be extracted with deep learning methods, and that these features can be successfully used for the classification of patients and controls.

For clinical practice, it would also be very useful to indicate for each classified subject the uncertainty of the classification. Related to this, one can consider predicting a scale rather than simply two classes, which would also better reflect the fact that many psychiatric disorders (including ASD) describe a spectrum rather than a binary diagnosis. Furthermore, ML methods can also be used for the prediction treatment responses. Hahn et al. (62), for instance, successfully predict treatment responses of patients with panic disorders by applying ML classifiers to fMRI data.

We discussed possible pitfalls and challenges that can occur during the classification pipeline. One such pitfall is double-dipping, i.e., the lack of separation of training and test set during feature selection. Double-dipping can markedly inflate the accuracy, especially for small and high-dimensional data sets.

Other challenges are more specific to the data sets commonly present when analyzing psychiatric disorders based on neuroimaging techniques, where the data are from multiple sites and often high-dimensional despite the data set being small in size. The underlying complexity of the disorder might encompass several diverse subtypes, and the high-dimensionality of this relatively small data set might easily lead to overfitting. This might explain why several of the presented out-of-the-box classifiers trump the accuracy of 60% from a proposed classifier specifically tailored for multisite ASD prediction (11, 12). Nevertheless, the classification results achieved are rather moderate, and it might be worthwhile to apply boosting and bagging [(24), Chapter 8]. Both techniques combine several weak learners (classifiers with moderate performance) to create a strong learner (a model with high classification performance). Boosting successively applies classifiers to the data set whereby more weight is given to data samples misclassified by the previous classifier. Boosting can reduce bias and variance. Bagging is the training of several weak learners with bootstrap samples of the original data as input for each learner. By taking several samples from the original data set and hence providing the classifier with more training data, bagging can reduce variance [(24), Chapter 8].

## Author Contributions

PF: main contribution in drafting the article, code implementation, data analysis and interpretation, as well as contributions to the modeling of data. CM and JB: contributions to the modeling of data, code implementation, data analysis and interpretation, and critical revision of draft. MM and NW: contributions to the modeling of data, data interpretation, and critical revision of draft. The article has been finally approved by all the authors, and accountability for any part of the article is taken by all the authors.

## Conflict of Interest Statement

The authors declare that the research was conducted in the absence of any commercial or financial relationships that could be construed as a potential conflict of interest.

## Code

The MATLAB code as discussed is available at http://www.ncm.hest.ethz.ch/downloads/software.html under “ABIDE 1 classification and pitfalls.”

## Appendix A1

### Preprocessing of the fMRI Data

Data presented in this study underwent standard fMRI preprocessing (i.e., realignment, normalization, and smoothing) implemented in SPM12b. Data were preprocessed according to standard SPM protocols including realignment, normalization to a study specific template using DARTEL, smoothing, band-pass filtering (0.01–0.05 Hz), and scrubbing to account for head movements (63). Specifically, structural images were first coregistered to the T1 template before the New Segmentation toolbox was used to segment the data into gray matter (GM), white matter (WM), and cerebrospinal fluid (CSF) images ready for input to the DARTEL toolbox (64). The DARTEL toolbox was used to create a study-specific template for normalization given that the average age of participants was in the range [10,14], and as such the Montreal Neurological Institute (MNI) template (generated using 18- to 30-year-old brains) would not be appropriate (65). Functional images were coregistered to the individual structural images, realigned, normalized to the MNI template space using DARTEL (resliced to 3 mm × 3 mm × 3 mm), and smoothed with an 8-mm kernel.

Adequately correcting for head motion artifact has proven to be an essential step in RS-fMRI analyses, especially in investigations of ASD (66, 67). Power et al. (63) and Van Dijk et al. (68) clearly demonstrated that even tiny movements, which are smaller than the spatial sampling size of fMRI data (i.e., movements >0.5 mm) have a large impact on studies of resting connectivity. In order to account for the confounding of micro-movements, an approach called scrubbing is applied to the data. Scrubbing involves removing bad data points where there is >0.5 mm framewise displacement (FD) or a >0.5% differential spatial variance (DVARS). We additionally modeled head movement using the Friston 24-parameter approach (69) to remove potential residual head motion signal (6 original regressors generated during realignment, 6 time-shifted regressors, and both of these squared) along with the first 3 principle component time series extracted from individual WM and CSF masks (70).

There are a number of atlases currently available for data reduction, and each atlas makes a different assumption about how to partition the cerebral cortex. Anatomical atlases like the Automated Anatomical Labeling (AAL) Atlas (71) or Harvard–Oxford Cortical Atlas are based either on gross morphological boundaries or on cytoarchitectonic features (see http://www.fz-juelich.de/inm/inm-1/EN/Forschung/_docs/SPMAnatomyToolbox/SPMAnatomyToolbox_node.html). However, atlases based on gross anatomy do not appropriately represent connectivity patterns seen in RS-fMRI data (72), probably because distinct functional subregions are grouped together, thus reducing sensitivity, and cytoarchitectonic atlases exist only for a few parts of the brain. Alternatively connectivity-based parcellations [CBP; (72–75)] and independent component analysis [ICA; (76, 77)] have been used to partition the cerebral cortex into voxels with common connectivity patterns. While, both CBP and ICA offer a useful middle ground between parcellations based on gross morphology and cytoarchitecture, one issue plaguing both methods is selecting how many regions/components to partition the data into. The Craddock atlas was chosen because it has been shown to outperform atlases based on gross morphology (15) and did not require us to arbitrarily choose thresholds necessary for CBP or ICA.

## Appendix A2

### Tests for Classification Significance

Statistical significance of classification can be assessed by means of permutation testing (47). The null hypothesis is that the classes do not differ. This can be tested by randomly permuting the labels of the data samples. The empirical *p*-value is then determined by the percentage of data sets with permuted labels where the classification delivers better results than on the original data. Ideally, all the data sets resulting from all possible label permutations should be used. Since this is usually not feasible due to the large number of possible permutations, an approximation of the size of randomized samples *k* necessary for a certain *p*-value *p* can be determined (78). Significance testing can also be performed with parametric tests where probability distributions are assumed, for instance, a binomial distribution for classification errors (a binomial distribution since a data sample can be either correctly classified or not). We refer the reader to Combrisson and Jerbi (53) who explain in detail how assuming a binomial distribution for the classification error, significant classification accuracies can be calculated with pre-implemented MATLAB functions for a given sample size.
